# Study on the relationship between lid-parallel conjunctival folds and symptoms and signs of dry eye disease

**DOI:** 10.3389/fmed.2025.1633155

**Published:** 2025-08-13

**Authors:** XiangTian Meng, Hongyan Zhou

**Affiliations:** China-Japan Union Hospital, Jilin University, Changchun, China

**Keywords:** lid-parallel conjunctival folds, LIPCOF, dry eye disease, conjunctival xerosis, tear film rupture time

## Abstract

**Purpose:**

The aim of this study is to explore the correlation between grade and total number of nasal and temporal side lid-parallel conjunctival folds (LIPCOFs) for signs and dry eye severity grading of different types of dry eye disease.

**Methods:**

The data of 76 eyes of 38 patients with dry eye disease were selected. Fluorescein Breakup Time (FBUT), tear secretion function, corneal staining test, and slit lamp examination were performed. The above results, including the grade and the total number of nasal and temporal side LIPCOFs, were recorded. The causal relationship between parametric variables was analyzed by Pearson’s correlation, and the causal relationship between non-parametric variables was analyzed by Spearman’s correlation. Paired *t*-test was used to analyze statistical differences between different groups. Benjamini-Hochberg False Discovery Rate (BH-FDR) correction is used to reduce the risk of type I error rate inflation when there is a correlation between multiple indicators.

**Results:**

A significant negative correlation was found between the grade of temporal side LIPCOF and the FBUT in all 76 eyes, both in the aqueous deficiency group and the non-aqueous deficiency group. Similarly, temporal side LIPCOF showed a reliable positive correlation with dry eye disease severity grading in the non-aqueous deficiency group. However, this correlation was not statistically significant in the aqueous deficiency group. For the grade of nasal side LIPCOF, there was no statistical correlation between the conjunctival fold grade and the FBUT in all 76 eyes. However, there was a significant positive correlation between the conjunctival fold grade and the FBUT in the aqueous deficiency group. In the non-aqueous deficiency group, there was a significant negative correlation between the conjunctival fold grade and FBUT. For the total number of LIPCOFs, there was no statistical correlation between the total number of LIPCOFs and FBUT in all 76 eyes and the aqueous deficiency group. However, there was a significant negative correlation between the total number of LIPCOFs and FBUT in the non-aqueous deficiency group.

## 1 Introduction

Dry eye disease, also known as xerophthalmia, manifests as dry, itchy eyes, foreign body sensation, photophobia, blurred vision, and other symptoms. It can cause serious damage to the health of the ocular surface. The prevalence rate in China is as high as 21 to 30%, which seriously affects people’s quality of life and reduces social productivity (1). The etiology of dry eye disease is complex and varied, including aqueous deficiency, lipid abnormalities, mucin abnormalities, and abnormal tear dynamics (2–4). The various factors are intricate and combine to contribute to the development of dry eye disease.

The nasal and temporal side LIPCOFs are parallel to the lower lid margins and are easily visualized by slit lamp examination; Causes include the presence of inflammation, decreased mucin, decreased elastic fibers, and increased friction during transient vision (5–9).

As with conjunctival laxity, the conjunctival folds are parallel to the lid margins in the tear stream, and both are thought to interfere with the tear stream (10–12). The result is an irregular tear film, but the effect on the tear film is more complex, with differences in the effect of the parallel nasal and temporal side LIPCOFs on the tear film (13). However, the different roles of nasal and temporal side LIPCOFs in different types of dry eye disease need to be investigated. Based on the above objectives, the relationship between the results of clinical tests and signs was analyzed in patients with dry eye disease of different etiologies.

### 1.1 Current status

Dry eye disease is a multifactorial ocular surface disease characterized by a loss of tear film homeostasis and a variety of ocular symptoms (2, 3). Its etiology includes tear film instability and hyperosmolarity, ocular surface inflammation and injury, and neurosensory abnormalities (2). Age, gender, geographic distribution, ocular surgery, use of video terminals, and incomplete transients may influence the onset of dry eye disease (14). The prevalence of dry eye disease is as high as 21 to 30% in China, which seriously affects people’s quality of life (1). The main components of the tear film on the ocular surface are the lipid layer, the aqueous layer, and the mucin layer, which distribute the tears on the ocular surface and eventually drain out of the eye through tear flow dynamics, such as blinking. A stable tear film is a sign of ocular health; it protects and moistens the cornea and is the first interface where light enters the visual system. Patients with dry eye disease primarily present with dry eyes, pain, itching, irritation, astringency, and foreign body sensation, and in severe cases, patients may develop corneal conjunctival lesions that can affect the patient’s vision.

### 1.2 Lid-parallel conjunctival folds

Lid-parallel conjunctival folds (LIPCOFs) are small bulbar conjunctival folds running parallel to the lower lid margin on the temporal and nasal sides of the bulbar conjunctiva. Studies have shown that patients with LIPCOF are more likely to develop dry eye disease (15, 16). LIPCOF also improves significantly after dry eye treatment (17, 18). Although LIPCOF may be the initial mild stage of conjunctival laxity, they have a slightly different clinical presentation. In addition, the cross-sectional area of LIPCOF is much smaller than that of conjunctival laxity (13). Conjunctival laxity can be induced or increased by forceful blinking and pressure on the lid margin. Conjunctival laxity can be induced or increased by forceful blinking and pressure on the lid margins, but LIPCOF are not affected by these factors (19). In contrast to conjunctival laxity, LIPCOF are often overlooked in the diagnosis of dry eye disease in clinical practice. In addition to the commonly used slit-lamp examination, studies have used anterior segment optical coherence tomography (OCT) to image the cross-sectional area of the LIPCOF and the extent to which they are covered by the Tear Meniscus (15, 20). There are no standardized grading criteria for LIPCOF, and some rely on the height of the folds compared to the height of the Tear Meniscus (21, 22), but the rationality of this grading has been questioned by many researchers (19, 23). In addition, there is a more predictive scheme based on the number of wrinkles (Table 1) proposed by Pult et al. (6, 24, 25). Their specific test consists of the patient blinking several times while looking straight ahead and the examiner observing the LIPCOF behind the temporal and nasal tear ducts of the lower eyelid and recording the number (26). During the examination, care must be taken to differentiate between true palpebral conjunctival folds: when the examiner pulls the lower eyelid away from the eye, true palpebral conjunctival folds disappear, but reappear after a blink and are always in the same position (27).

**Table 1:** LIPCOFs grading criteria (6, 24, 25).

Clinical signs	LIPCOF grade
No conjunctival folds or disrupted microfolds in one line	0
One permanent and clear parallel fold	1
Two permanent and clear parallel folds (normally lower than 0.2 mm)	2
More than two permanent and clear parallel folds (normally higher than 0.2 mm)	3

### 1.3 Causes and effects

#### 1.3.1 Causes of LIPCOF

Relatively few studies have been conducted on the causes of LIPCOF: (1) the same etiology as conjunctival chalasis (CCh) (28, 29), including laxity of the organization of the bulbar conjunctiva and sclera, conjunctival thinning, decreased elasticity, and decreased turgor (30); and (2) an increase in friction and frequency of friction during the transient eye, including insufficient lacrimal tears, decreased mucin production, tear film viscosity, transient eye frequency, incomplete transients, and brush epithelial lesions (6–9, 24).

#### 1.3.2 Role of LIPCOF in dry eye disease pathogenesis

**Figure 1:**
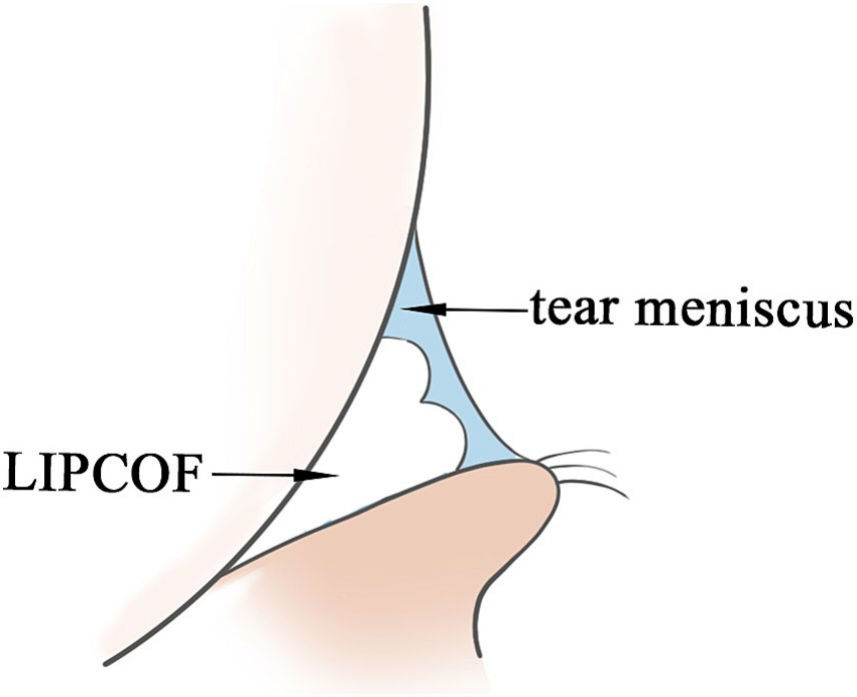
Schematic of the tear meniscus and LIPCOF cross-section.

As with genesis, the role of LIPCOF in the pathogenesis of dry eye disease has been less studied. Tears on the ocular surface are located in three main areas: in the sclera and cornea covering the exposed region of the lid fissure, in the canthal tear stream, and in the conjunctival sac of the upper and lower eyelids. Of these, the lid margin tears account for 75 to 90% of the total volume of tears to the ocular surface, and LIPCOF may affect the volume of the tear meniscus and the stability of the tear meniscus (10–13): LIPCOF occur behind the temporal and nasal tear meniscus and can account for up to two-thirds of the total length of the tear meniscus (31), and the height of the tear meniscus in the central position of the eyelids is also influenced by LIPCOF. Under fluorescein staining, weak or even no fluorescence was observed at LIPCOF compared to the tear meniscus of subjects without LIPCOF, suggesting that the tear film layer is too thin at this location (15). It is well visualized by anterior segment OCT that the conjunctival folds interfere with the tear fluid in the tear stream, disrupting the original crescent-shaped appearance of the tear stream, and the actual cross-sectional area of the tear stream is reduced (Figure 1) (20). It has been hypothesized that this also leads to a shift to the central portion of the tear film. One study showed that patients with an increased degree of LIPCOF tended to have an effect on the height of the central tear flow: while temporal side LIPCOF affected the height of the central tear flow at the upper and lower lid margins, nasal side LIPCOF affected the height of the tear flow at the lower lid only (13). However, it has also been suggested that the influence of LIPCOF on central tear level may also be due to the obstructive effect of LIPCOF on tear outflow as well as capillary action (31). Central tear film height is an important assessment in the diagnosis of dry eye disease (32), but may be misclassified in patients with LIPCOF.

#### 1.3.3 Similarities and differences between nasal and temporal side LIPCOFs in the development of dry eye disease

According to the lacrimal flow model developed by Professor Lee of the Department of Mathematical Sciences at the University of Delaware (33), there is a pressure gradient within the lacrimal flow that drives fluid away from the lacrimal region and ultimately out through the lacrimal point. The inferior tear stream drains directly through the inferior canthus, and the superior tear stream may bypass the lateral canthus and enter the inferior tear stream in addition to draining through the superior canthus (33). This may also be the reason why temporal side LIPCOF affects the height of the upper tear meniscus, whereas nasal side LIPCOF does not affect the height of the upper tear meniscus (13). Tear film stability is one of the reasons for the development of dry eye disease, and decreased volume, irregularity or discontinuity of the tear film, and abnormal tear drainage may lead to poor tear film formation (31, 34, 35). Imbalance in tear film stability is mainly characterized by shortened Fluorescein Breakup Time (FBUT) and altered tear film morphology (36).

**Figure 2:**
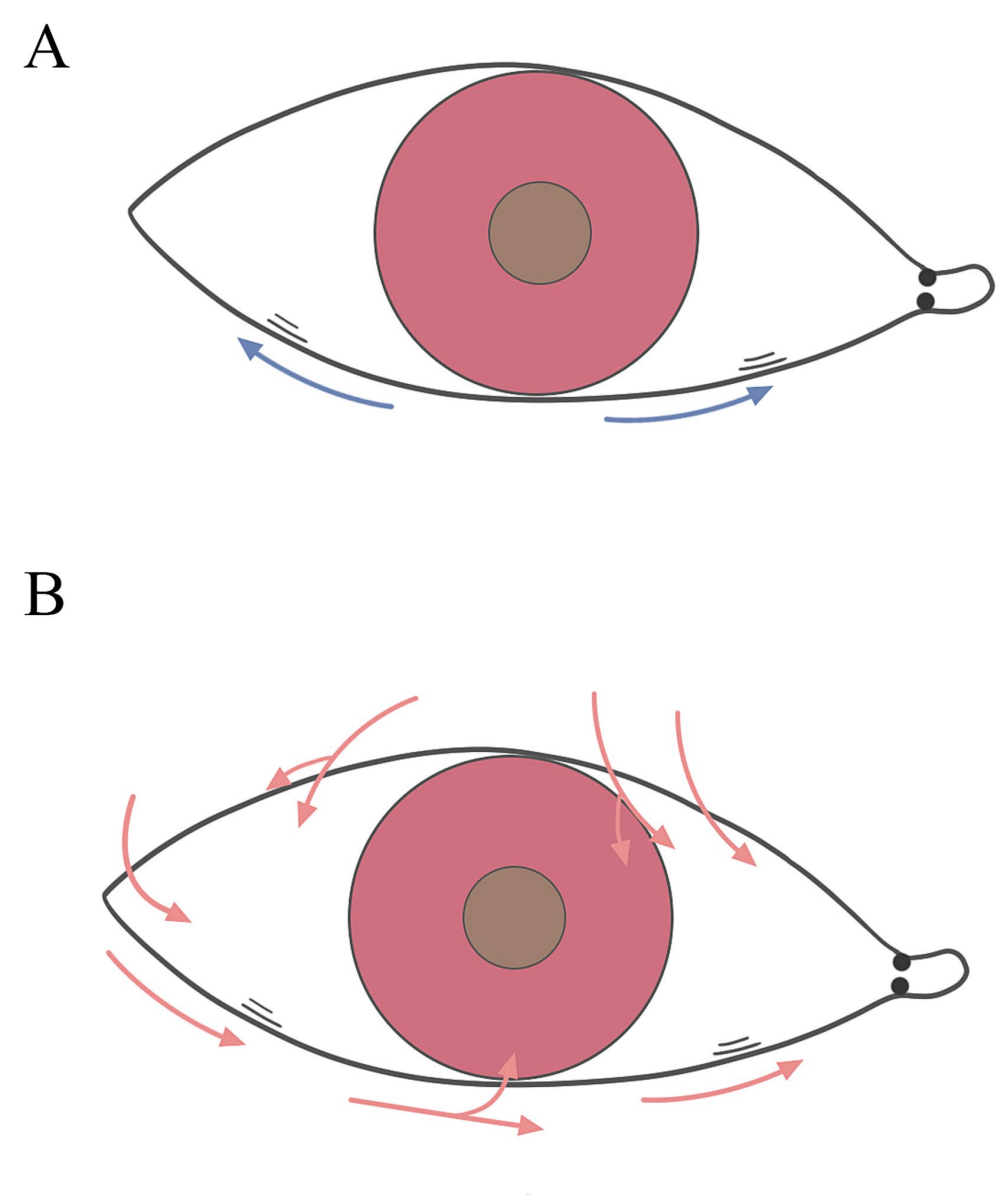
Role of nasal and temporal side LIPCOFs for the tear meniscus. **(A)** The blue arrows represent the capillary effect. **(B)** The red arrows represent the direction of tear flow.

Therefore, the role of nasal and temporal side LIPCOFs in the development of dry eye varies (Table 2). In addition to reducing the actual volume of the tear film at the respective location, both nasal and temporal side LIPCOFs may influence the height of the central tear film. The effect of LIPCOF on the height of the central tear film in the lower lid consists of capillary forces generated between the conjunctival folds that draw tears toward the folds, and this capillary effect may be stronger when more than one conjunctival fold is present (Figure 2A). LIPCOF can also be considered a barrier to the normal flow of tears along the lid (5, 37), and due to the previously mentioned direction of tear flow (33), the barrier effect of LIPCOF on the nasal and temporal sides is different for the tear flow in the middle of the lower lid (Figure 2B). The nasal side LIPCOF obstructs the flow of tears into the lower tear duct, which in turn delays tear drainage; the temporal side LIPCOF obstructs the entry of tears into the central tear duct of the lower eyelid, which increases the irregularity of the tear duct. And because the nasal tear flow is not directly connected to the upper lid tear flow, unlike temporal side LIPCOF, which affects the height of both the upper and lower lid tear flows, nasal side LIPCOF only affects the height of the lower lid tear flow. However, both can ultimately cause irregularities and discontinuities in the tear film and a reduction in the actual volume of the tear film. Therefore, nasal side LIPCOF do not have the same degree of predictability in the diagnosis of dry eye disease as those parallel to the temporal lid margin (7, 24).

**Table 2:** Comparison of nasal and temporal side LIPCOFs action.

Indicators	Temporal side LIPCOF	Nasal side LIPCOF
Tear flow in the middle of the lower lid	1. Capillary effect 2. Slowing down the drainage of the Tear Meniscus	1. Capillary effect 2. Slowing down the flow of tears into the lower central tear river, aggravating the irregularity of the Tear Meniscus
Tear flow in the middle of the upper lid	No affection	1. Capillary action 2. Slowing down the drainage of the Tear Meniscus
Other	Decrease in the volume of the actual Tear Meniscus after occupying the Tear Meniscus	Decrease in the volume of the actual Tear Meniscus after occupying the Tear Meniscus

#### 1.3.4 LIPCOF and different types of dry eye disease

The Tear Film and Ocular Surface Society (TFOS) Dry Eye Workshop (DEWS) states that subcategories of dry eye can be divided into dry eye disease with predominantly excessive evaporation, dry eye disease with predominantly aqueous deficiency, and a greater role for both (2). The former refers primarily to inadequate tear production, while the latter includes conditions such as lipid layer abnormalities as well as increased tear evaporation due to transient imperfections (2). Patients with significantly reduced tear production (i.e., Schirmer I test ≤10 mm/5 min) were considered to have aqueous deficiency dry eye disease, while the remainder were considered to have non-aqueous deficiency dry eye disease.

Both aqueous deficiency and hyper-evaporative dry eyes may play a role in the etiology of LIPCOF, but the altered tear dynamics caused by LIPCOF is a factor in hyper-evaporation, which is not the main causative factor in aqueous deficiency dry eyes. Therefore, the correlation between LIPCOF and aqueous deficiency dry eyes and non-aqueous deficiency dry eyes may be different (38).

### 1.4 Conclusion

The correlation between LIPCOF, an easily observed but equally easily missed clinical sign, and the development of dry eye disease is well established. However, nasal and temporal side LIPCOFs and the presence or absence of aqueous deficiency may influence the correlation between LIPCOF and the development of dry eye disease. Therefore, further study of LIPCOF may provide a better understanding of the influence of different factors in the development of dry eye disease and provide new ideas for the diagnosis, classification, grading, and outcome evaluation of dry eye disease.

## 2 Experimental materials and methods

### 2.1 Subjects

A total of 38 dry eye disease patients (76 eyes in total; mean age 47.58 years; including 9 men and 29 women) from the outpatient ophthalmology clinic of the China-Japan Friendship Hospital of Jilin University were selected, and all these dry eye disease patients with typical dry eye disease symptoms were included in the analysis. All examinations were performed on the same day for each patient. Patients with ocular surface diseases other than dry eye, younger than 18 years of age, with a history of ocular surgery, including refractive surgery, eyeliner tattooing, eyelid surgery, or corneal surgery; any ocular trauma; diabetes mellitus; medication or use of medications known to affect the ocular surface and/or tear film; contact lens wear within 2 weeks; pregnancy; and conjunctival laxity (defined as a condition in which blinking forcefully or applying pressure with a finger to the edge of the eyelid causes the conjunctival folds to widen, or the presence of at least one central conjunctival fold) are all excluded. All procedures were performed in accordance with the Declaration of Helsinki (1983), all subjects provided written informed consent before participating in the study, and patient data were anonymized.

### 2.2 Test materials

This study included the following materials: saline solution (Jilin Kangnaier Pharmaceutical Co.); sodium fluorescein ophthalmic test strips (Liaoning Meizilin Pharmaceutical Co.); and lacrimal secretion test filter paper strips (Tianjin Inoxin Kang Medical Device Technology Co.).

### 2.3 Experimental instruments

This study used the Slit lamp microscope (Zeiss SL130, Germany) for the experiments.

### 2.4 Experimental procedure

#### 2.4.1 Grading of LIPCOF

**Figure 3:**
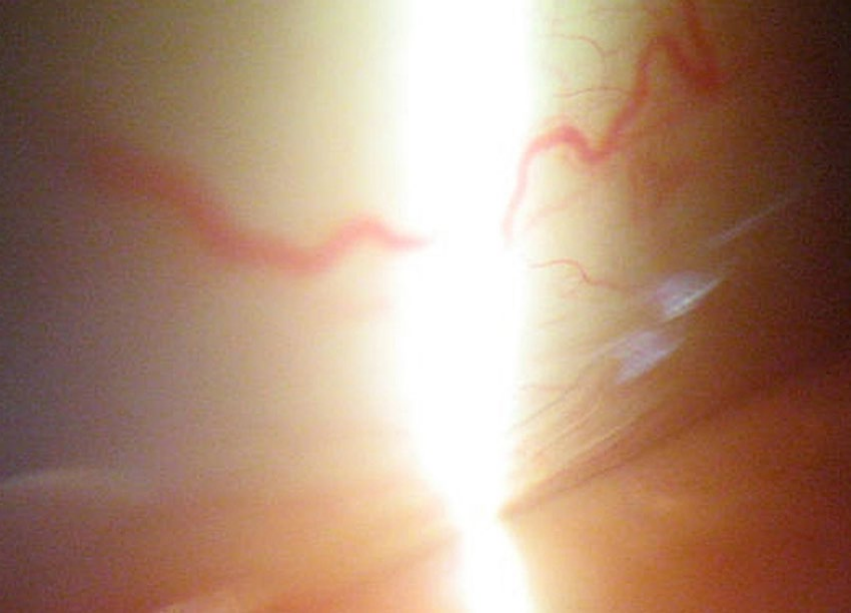
LIPCOF Grade 2 (Taken by Zeiss SL130, image with consent of subject).

The test is performed in a room at room temperature, protected from light, and with appropriate humidity. The patient is asked to look straight ahead and blink several times. The examiner looks for horizontal conjunctival folds from the center of the lower lid to the nasal and temporal third (26). The number of nasal and temporal side LIPCOFs and their sum were recorded separately (Figure 3). During the examination, care must be taken to distinguish true LIPCOF: when the examiner pulls the lower eyelid away from the eye, true LIPCOF disappear, but they reappear after a blink and are always in the same position (27). Nasal and temporal side LIPCOFs were graded according to the method described by Plut et al. (Table 1).

#### 2.4.2 Tear test (Schirmer I test)

The test was performed in a room at room temperature, protected from light, and with appropriate humidity. Schirmer’s test paper (5 mm × 35 mm) was used, with the head end folded inwards and placed in the conjunctival sac at the junction of the outer middle third of the lower eyelid. The patient was instructed to gently close the eyes and turn the eyeball slightly upward. After 5 min, the eyelid was gently pulled down, the strip of filter paper was removed, and the scale of the tear-soaked test paper was read. The Schirmer’s I test is performed without anesthesia and reflects the secretory function of the main lacrimal gland (35).

#### 2.4.3 FBUT

It was performed at room temperature, protected from light, and with appropriate humidity. Saline was used to moisten the fluorescein test paper to avoid excessive drug residue on the fluorescein test paper. Using the moistened fluorescein paper to wet the lower lid margins, the patient blinked three to four times to coat the ocular surface with fluorescein, and the eyes were viewed flat under the cobalt blue light of a slit lamp microscope. The time from the last transient to the appearance of the first black spot on the cornea, i.e., the FBUT, was calculated and recorded, measured three times, and averaged.

#### 2.4.4 Fluorescein corneal staining

This was performed at room temperature, protected from light, and in a room with appropriate humidity. After fluorescein staining, green spots were observed under the cobalt blue light of a slit lamp microscope, and the extent of staining, number, and presence of fusions or filaments were recorded.

#### 2.4.5 Dry eye typing

Patients with significantly reduced tear production (i.e., Schirmer I test ≤ 10 mm/5 min) were included in the aqueous deficiency group. The remainder were in the non-aqueous deficiency group.

#### 2.4.6 Dry eye severity grading

According to the Chinese Dry Eye Expert Consensus (35), dry eye can be categorized according to the severity of the signs as follows: Mild, FBUT of 2 s or more, no obvious ocular surface damage (i.e., <5 corneal fluorescein staining spots) on slit-lamp examination; Moderate: FBUT of 2 s or more, with corneal damage extending to no more than 2 quadrants and/or corneal fluorescein spots ≥5 but <30 on slit-lamp examination; Severe: FBUT <2 s; slit-lamp examination of corneal damage extending over 2 quadrants and beyond, and/or ≥30 corneal fluorescein spots; corneal fluorescein spots appearing to be fused or accompanied by filaments. Mild, moderate, and severe dry eye were scored 1, 2, and 3 points, respectively. A score of 0 was given for not meeting the diagnostic criteria for dry eye.

#### 2.4.7 Dry eye diagnostic criteria

1. Patients complained of subjective symptoms, such as dryness, foreign body sensation, burning, fatigue, discomfort, redness, fluctuation of vision, etc.; at the same time, patients with FBUT ≤5 s or Schirmer I test ≤5 mm/5 min could be diagnosed with dry eye. 2. At the same time, patients with FBUT >5 s and ≤10 s or Schirmer I test >5 mm/5 min and ≤10 mm/5 min had dry eye-related symptoms. The corneal conjunctiva must be examined by sodium fluorescein staining, and positive staining (≥5 spots) can diagnose dry eye.

### 2.5 Statistical methods

SPSS23.0 statistical software was used to process the data collected in this experiment, and the causal relationship between parametric variables was analyzed by Pearson’s correlation, and the causal relationship between non-parametric variables was analyzed by Spearman’s correlation. Paired t-test was used to analyze statistical differences between different groups. BH-FDR correction is used to reduce the risk of type I error rate inflation when there is a correlation between multiple indicators.

## 3 Results

In a total of 76 eyes, 35 eyes had Schirmer I test equal to or less than 10 mm/5 min (aqueous deficiency group), and 41 eyes had Schirmer I test greater than 10 mm/5 min (non-aqueous deficiency group). Patients in the aqueous deficiency group had a shorter FBUT and a higher dry eye severity score than those in the non-aqueous deficiency group. Moreover, due to the difference in the prevalence of different types of dry eye at different ages (39), the mean age of the aqueous deficiency group was significantly higher than that of the non-aqueous deficiency group. However, there was no statistically significant difference between the nasal and temporal side LIPCOF scores and the total number of subjects in both groups. The proportion of women in both groups was close (77.1% vs. 78.0%), *p* = 0.920, suggesting a balanced gender distribution, which is consistent with the epidemiological profile of the high prevalence of dry eye in women (14) (Table 3).

**Table 3:** Baseline characteristics.

Indicators	Aqueous deficiency group (*n* = 35)	Non-aqueous deficiency group (*n* = 41)	Total	Statistical difference (*p*-value)
Gender (Eyes from women)	27 (77.1%)	32 (78.0%)	59 (77.6%)	0.920
Age	51.83 ± 10.813	43.95 ± 16.571	47.58 ± 14.665	0.015
Temporal side LIPCOF grade	1.49 ± 0.658	1.59 ± 0.774	1.54 ± 0.72	0.551
Nasal side LIPCOF grade	0.83 ± 1.014	1.10 ± 0.917	0.97 ± 0.966	0.229
Number of LIPCOFs	2.31 ± 1.105	2.63 ± 1.280	2.49 ± 1.205	0.252
FBUT	2.283 ± 1.457	3.883 ± 1.781	3.146 ± 1.816	< 0.001
Schirmer I test	1.69 ± 0.900	1.07 ± 0.721	1.36 ± 0.860	0.002

A total of 38 patients were included, 9 men and 29 women, with a mean age of (47.58 ± 14.665) years. The demographic data and the mean and standard deviation of the results of the different tests are shown in Table 4. There were no significant differences between the nasal and temporal side LIPCOF scores and the total number of LIPCOFs, FBUT, tear secretion function measurements, and dry eye severity grading between the left and right eyes (Table 5).

**Table 4:** Correlation between ocular laterality and clinical signs.

Indicators	Right eye	Left eye	Total	Statistical difference (*p*-value)
Temporal side LIPCOF grade	1.53 ± 0.725	1.55 ± 0.724	1.54 ± 0.72	0.875
Nasal side LIPCOF grade	1.05 ± 1.038	0.87 ± 0.906	0.96 ± 0.972	0.412
Number of LIPCOFs	2.53 ± 1.39	2.42 ± 1.03	2.47 ± 1.216	0.709
FBUT	3.066 ± 1.7068	3.226 ± 1.9389	3.146 ± 1.8161	0.703
Schirmer I test	13.16 ± 7.807	13.79 ± 8.647	13.47 ± 8.189	0.739
Dry eye severity grading	1.39 ± 0.887	1.32 ± 0.842	1.36 ± 0.860	0.692

**Table 5:** Correlation between sex and clinical signs.

Indicators	Male	Female	Total	Statistical difference (*p*-value)
Number of people	9	29	38	
Age	44.89 ± 16.23	48.41 ± 14.193	47.58 ± 14.665	0.377
Temporal side LIPCOF grade	1.61 ± 0.502	1.52 ± 0.778	1.54 ± 0.72	0.551
Nasal side LIPCOF grade	1.11 ± 1.023	0.91 ± 0.96	0.96 ± 0.972	0.456
Number of LIPCOFs	2.61 ± 1.092	2.43 ± 1.258	2.47 ± 1.216	0.587
FBUT	3.889 ± 1.6467	2.916 ± 1.8173	3.146 ± 1.8161	0.046
Schirmer I test	14.83 ± 8.403	13.05 ± 8.149	13.47 ± 8.189	0.424

Age, nasal and temporal side LIPCOFs and their total number, and tear secretion function measurements were not significantly different between the sexes. However, FBUT was not statistically significantly different between the sexes (*p*-value = 0.046) (Table 6).

**Table 6:** Correlation between LIPCOF and clinical signs.

Indicators		Temporal side LIPCOF grade	Nasal side LIPCOF grade	Number of LIPCOFs
Age	Correlation coefficient	0.159	−0.069	0.029
	Significance	0.169	0.556	0.803
FBUT	Correlation coefficient	−0.381*	0.065	−0.190
	Significance	0.001	0.580	0.100
Schirmer I test	Correlation coefficient	−0.001	0.216	0.144
	Significance	0.993	0.061	0.215
Dry eye severity grading	Correlation coefficient	0.246	−0.197	−0.002
	Significance	0.032	0.087	0.987

*Significant results after BH-FDR correction (*p* = 0.05).

Neither the nasal and temporal side LIPCOFs scores nor their total number showed a significant correlation with age, Schirmer I test, and dry eye severity grading. The temporal side LIPCOF scores showed a negative correlation with FBUT; however, the nasal side LIPCOF scores and the total number of LIPCOFs showed no correlation with FBUT (Table 6).

In all subject eyes, the temporal side LIPCOF showed a significant negative correlation with FBUT. Similarly, temporal side LIPCOF showed a similar negative correlation in both aqueous and non-aqueous groups, but this negative correlation was more reliable in the non-aqueous group. Similarly, temporal side LIPCOF showed a reliable positive correlation with dry eye severity grading in the non-aqueous deficiency group. However, this correlation was not statistically significant in the aqueous deficiency group (Table 7).

**Table 7:** Correlation between LIPCOF and clinical signs in Aqueous deficiency group and Non-aqueous deficiency group.

Indicators		Temporal side LIPCOF grade	Nasal side LIPCOF grade	Number of LIPCOFs
Aqueous deficiency group (35)				
FBUT	Correlation coefficient	−0.423*	0.519*	0.224
	Significance	0.011	0.001	0.195
Dry eye severity grading	Correlation coefficient	0.166	−0.351	−0.223
	Significance	0.341	0.039	0.198
Non-aqueous deficiency group (41)				
FBUT	Correlation coefficient	−0.482*	−0.394*	−0.583*
	Significance	0.001	0.011	0.000
Dry eye severity grading	Correlation coefficient	0.414*	0.065	0.301
	Significance	0.007	0.688	0.056

*Significant results after BH-FDR correction (*p* = 0.05).

In contrast to the temporal side LIPCOF, the nasal side LIPCOF showed diametrically opposite effects in both the aqueous deficiency and non-aqueous deficiency groups. There was no reliable correlation between the grading of nasal side LIPCOF and FBUT in any of the subject eyes. However, in the aqueous deficiency group, the nasal side LIPCOF showed a reliable positive correlation with FBUT, whereas in the non-aqueous deficiency group, it showed a reliable negative correlation. Whereas nasal side LIPCOF in the aqueous deficiency group and the non-aqueous deficiency group showed no statistical correlation with dry eye severity grading (Table 7).

The total number of LIPCOFs showed a positive correlation with FBUT only in the non-aqueous deficiency group. There was no statistical correlation between the others and the indicators (Table 7).

## 4 Discussion

There was no significant correlation between age and LIPCOF in any of the eyes in this experiment. However, in the aqueous deficiency group, the temporal side LIPCOF grading showed a positive correlation with age. Previously, it was thought that the correlation between LIPCOF grading and age may be due to this age-related degeneration and may also be related to the fact that dry eye is more common in the older age group (40). Thus, in the present study, the higher mean age of the aqueous deficiency group may support the latter idea. There was no correlation between LIPCOF and gender.

**Figure 4:**
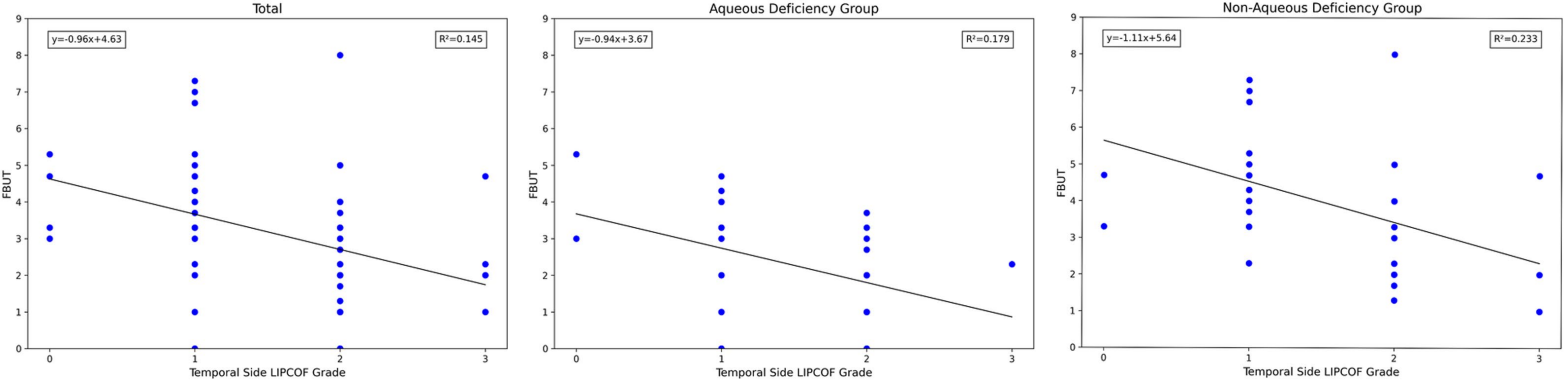
Scatterplot of temporal side LIPCOFs grading correlating with FBUT.

For the temporal side LIPCOF, there was a significant correlation between the grade of temporal side LIPCOF and objective signs of dry eye in all subject eyes, in the aqueous deficiency group, and in the non-aqueous deficiency group (Figure 4).

The higher the grade of temporal side LIPCOF, the shorter the FBUT. This correlation was slightly stronger and more pronounced in the non-aqueous deficiency group.

**Figure 5:**
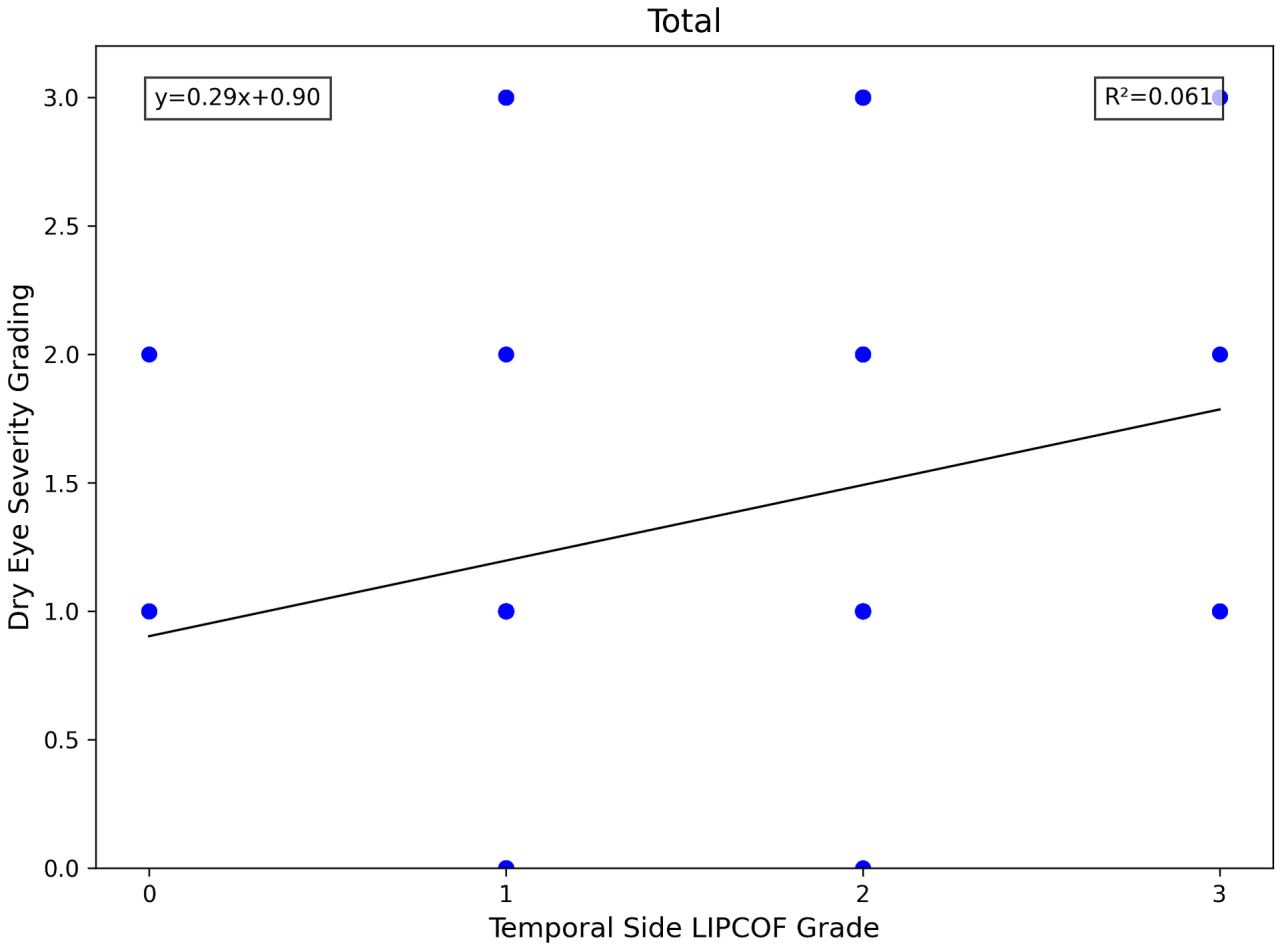
Scatterplot of temporal side LIPCOFs grading correlating with dry eye severity grading.

Similarly, temporal side LIPCOF was associated with dry eye severity grading only in the non-aqueous deficiency group (Figure 5), but not in the aqueous deficiency group. This is due to the fact that the pathogenesis of aqueous deficiency dry eye is mainly due to decreased tear secretion (2), so dry eye severity grading did not show an association with LIPCOF in the aqueous deficiency group.

**Figure 6:**
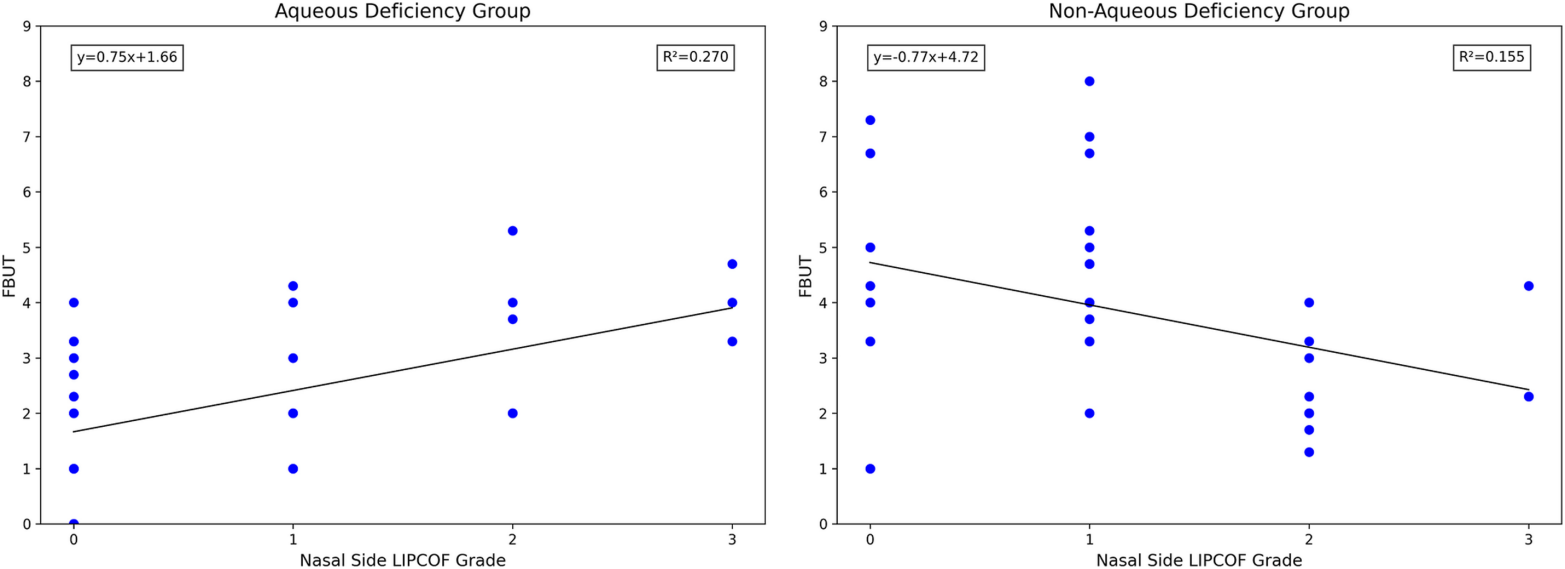
Scatterplot of nasal side LIPCOFs grading correlating with FBUT.

Notably, unlike the grade of temporal side LIPCOF, the grade of nasal side LIPCOF and objective signs of dry eye showed diametrically opposite results in the aqueous versus non-aqueous deficiency groups (Figure 6). In the non-aqueous deficiency group, similar to the effect of temporal side LIPCOF, the higher the grade of nasal side LIPCOF, the shorter the FBUT. However, in the aqueous deficiency group, the nasal side LIPCOF was positively correlated with FBUT.

This difference may be explained by two different mechanisms of nasal and temporal side LIPCOFs in the pathogenesis of dry eye (Table 2). First of all, the “capillary effect” produced by the parallel nasal and temporal side LIPCOFs and their own changes to the actual volume of the Tear Meniscus lead to a decrease in the actual volume of the Tear Meniscus and irregularity of the Tear Meniscus, resulting in a shortened FBUT (10–13, 31). At the same time, the conjunctival fold parallel to the palpebral margin can act as a barrier to tear flow (5, 37). However, due to the direction of tear drainage (33), this “barrier” prevents the smooth flow of tears into the central lacrimal river due to the parallel temporal side LIPCOF, which aggravates the instability of the tear film. In the case of nasal side LIPCOF, this “barrier” slows down the flow of tears into the puncta and reduces tear drainage. In the aqueous deficiency group, the “barrier” effect of nasal side LIPCOF may have contributed to a delay in tear drainage, resulting in a delay in tear film rupture. However, in the non-aqueous deficiency group, the effect of this delay in tear discharge led to irregularities in the tear film, which led to a shortening of the FBUT. Of course, this effect is limited, and when the tear secretion is low enough, nasal side LIPCOF will have little effect on the FBUT. The effect of nasal side LIPCOF on dry eye may have a cutoff point based on tear secretion capacity. If the tear secretion capacity is lower than a certain value, nasal side LIPCOF has an inhibitory effect on the stability of the tear film. Above this value, nasal side LIPCOF mainly promotes tear film stability.

**Figure 7:**
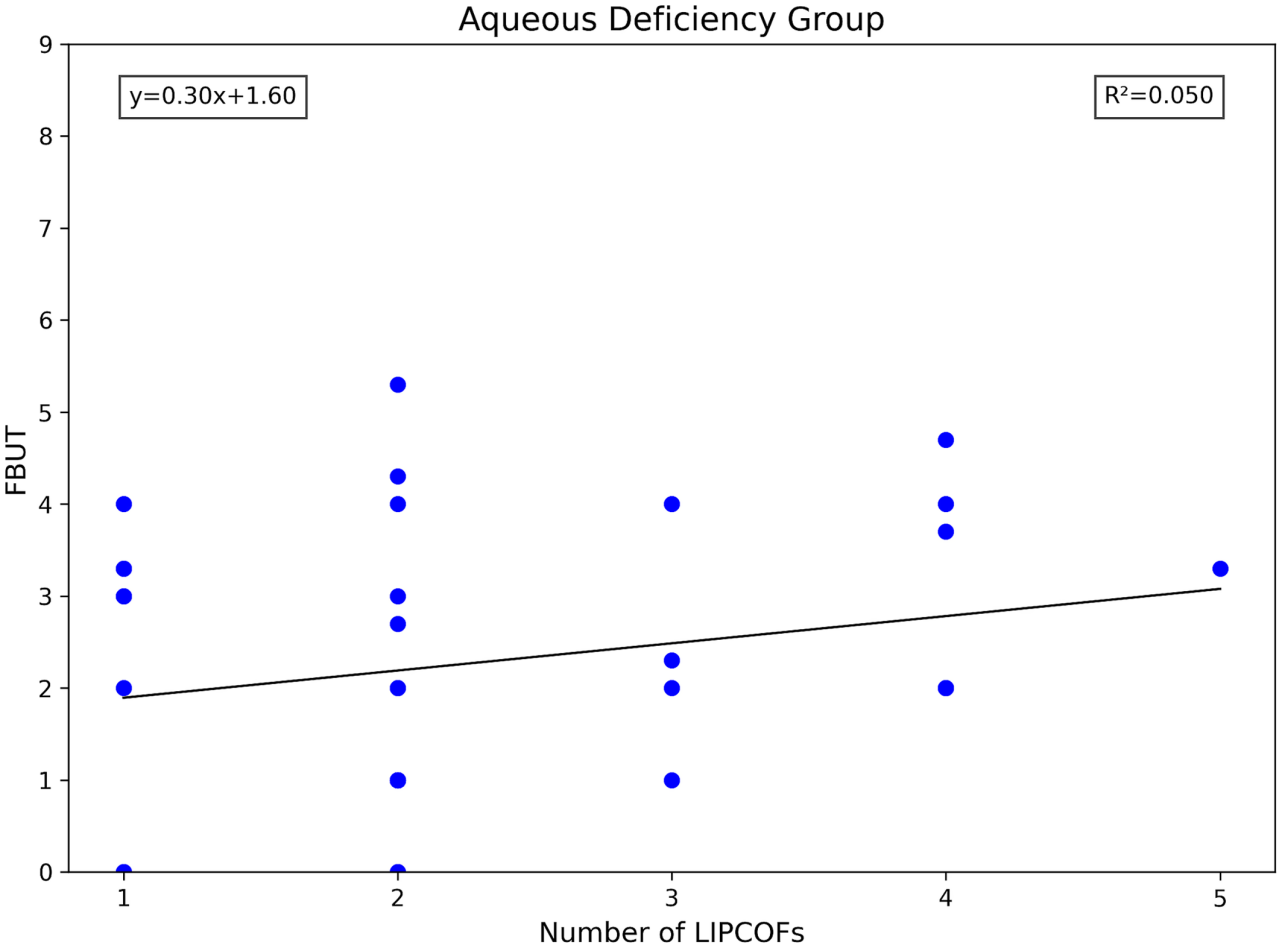
Scatterplot of number of LIPCOFs correlating with FBUT.

The total number of LIPCOFs only showed a positive correlation with FBUT in the non-aqueous deficiency group. However, compared with the grade of temporal and nasal side LIPCOFs, the correlation is stronger and the significance is higher (Figures 4, 6, 7).

As with other classic diagnostic tests for dry eye, correlations between LIPCOF and other diagnostic findings are not high (7, 41). In fact, these relatively low correlations are common because different diagnostic tests reflect different and relatively independent anatomy, physiology, or pathology of the tear film and ocular surface. Therefore, I believe that the low level of correlation between the results of different diagnostic tests does not represent the inapplicability of individual tests, but rather reflects the multiple etiologies and different pathophysiological mechanisms involved in the complex phenomenon of dry eye. The results of the present experiment demonstrate that LIPCOF also behaves relatively independent in the pathogenesis of dry eye, as it shows a statistically significant correlation of moderate strength with dry eye and disease severity, but not with other classic dry eye diagnostic tests. Dry eye is a multifactorial disease with many different subtypes in terms of origin and pathomechanism (42). A good combination of different tests may cover a wider range of dry eye diagnoses. Further studies should attempt to establish the relationship between the etiology and pathological type of dry eye and the grade of LIPCOF to understand the genesis and role of LIPCOF.

In conclusion, the results of this study show that the nasal side LIPCOF can have different effects due to different tear secretion functions. In future, experiments can be further designed to study the mechanism of the LIPCOF effect based on this feature.

The total number of LIPCOFs showed a positive correlation with FBUT only in the non-aqueous deficiency group, which can be used as a primary screening method for tear film stability in patients with non-aqueous deficiency dry eye to reduce invasive procedures.

Temporal side LIPCOF showed moderate sensitivity and specificity for tear film stability, both in aqueous and non-aqueous deficiency groups. What is more surprising is that the temporal side LIPCOF shows dry eye severity grading, which is very suitable as a preliminary screening method for dry eye diagnosis and the basis for preliminary judgment of severity, so as to better guide further diagnosis and treatment. ROC analysis was performed, reporting the following key metrics:

- AUC (Area Under the Curve): 0.692 (95% CI: 0.571–0.813).
- Optimal Cutoff Value: LIPCOF ≥1.5 (corresponding to grade ≥2).
- Sensitivity: 78.3% (95% CI: 65.3–87.5%).
- Specificity: 54.3% (95% CI: 41.8–66.3%).

An AUC of 0.692 (indicating moderate diagnostic value) demonstrates that temporal LIPCOF grading has significant discriminatory power for moderate-to-severe dry eye (grade ≥2). The Youden index was maximized at LIPCOF ≥1.5 (cutoff value 1.5, corresponding to grade ≥2):

- Sensitivity 78.3%: This indicates good ability to correctly identify true moderate-to-severe dry eye patients.
- Specificity 54.3%: This indicates a certain level of false positives; individuals with LIPCOF ≥1.5 (grade ≥2) are recommended to undergo supplementary tests, including tear film breakup time (BUT) and corneal fluorescein staining, to confirm the diagnosis.

But it is worth noting that the relatively small sample size in subgroups may limit statistical power. However, BH-FDR correction has been used in studies to reduce the risk of type I error rate inflation and improve reliability.

Given its non-invasive and simple nature, I suggest the grade of LIPCOF be used as an additional rapid dry eye screening test and as part of a routine in ophthalmic clinical examination protocol, as well as an evaluation of effectiveness. At the same time, test panels can be constructed to facilitate dry eye classification and improve prediction.

## 5 Conclusion

In this cohort study, the nasal/temporal side LIPCOF grade scale and total Number of LIPCOFs were validated for correlation with tear film stability and dry eye severity grading in patients with aqueous and non-aqueous deficiency dry eye, respectively. The results showed that temporal side LIPCOF was an important differentiator of all types of dry eye syndrome and could well reflect the dry eye severity grading. Nasal side LIPCOF exhibits diametrically opposed effects in patients with aqueous and non-aqueous deficiency dry eye. The total number of LIPCOFs showed a significant positive correlation with tear film stability in the non-aqueous deficiency group.

## Data Availability

The original contributions presented in the study are included in the article/Supplementary material, further inquiries can be directed to the corresponding author.
